# Gamma-delta T cells stimulate IL-6 production by pancreatic stellate cells in pancreatic ductal adenocarcinoma

**DOI:** 10.1007/s00432-020-03367-8

**Published:** 2020-08-31

**Authors:** Adrian M. Seifert, Julian List, Max Heiduk, Rahel Decker, Janusz von Renesse, Ann-Christin Meinecke, Daniela E. Aust, Thilo Welsch, Jürgen Weitz, Lena Seifert

**Affiliations:** 1 Department of Visceral, Thoracic and Vascular Surgery, University Hospital Carl Gustav Carus, Medical Faculty, Technische Universität Dresden, 01307 Dresden, Germany; 2 German Cancer Research Center (DKFZ), German Cancer Consortium (DKTK), Partner Site Dresden, Heidelberg, Germany; 3 National Center for Tumor Diseases (NCT), Partner Site Dresden, German Cancer Research Center (DKFZ), Heidelberg, Germany; 4 Department of Pathology, Medical Faculty, University Hospital Carl Gustav Carus, University of Dresden, Dresden, Germany; 5 NCT Biobank Dresden, University Hospital Carl Gustav Carus, Technische Universität Dresden, Dresden, Germany

**Keywords:** Pancreatic cancer, Gamma-delta T cells, Pancreatic stellate cells, IL-6

## Abstract

**Introduction:**

The immunosuppressive tumor microenvironment promotes progression of pancreatic ductal adenocarcinoma (PDAC). γδ T cells infiltrate the pancreatic tumor stroma and support tumorigenesis through αβ T cell inhibition. Pancreatic stellate cell (PSC) activation contributes to pancreatic fibrosis in PDAC, limiting the delivery and efficacy of therapeutic agents. Whether γδ T cells have direct effects on PSC activation is unknown.

**Methods:**

In this study, we analyzed tumor tissue from 68 patients with PDAC and determined the frequency and location of γδ T cells using immunohistochemistry and immunofluorescence. PDAC samples from the TCGA database with low and high *TRGC2* expression were correlated with the expression of extracellular matrix genes. Further, PSCs were isolated from pancreatic tumor tissue and co-cultured with γδ T cells for 48 hours and cytokine production was measured using a cytometric bead array.

**Results:**

γδ T cells infiltrated the pancreatic tumor stroma and were located in proximity to PSCs. A high infiltration of γδ T cells was associated with increased expression of several extracellular matrix genes in human PDAC. In vitro, γδ T cells stimulated IL-6 production by PDAC-derived PSCs.

**Conclusion:**

γδ T cells activated PSCs and modulation of this interaction may enhance the efficacy of combinational therapies in human PDAC.

## Introduction

Pancreatic ductal adenocarcinoma (PDAC) is an aggressive tumor and projected to become the second leading cause of cancer-related deaths by 2030 (Yadav and Lowenfels 2013). One of the main reasons for its poor prognosis is the extensive fibrotic tumor stroma that impedes successful cancer therapies. Studies employing checkpoint inhibitors in PDAC have failed to elicit a sufficient response, likely due to immunosuppression mediated by the tumor stroma (Brahmer et al. 2012). The tumor stroma, harboring immune cells and pancreatic stellate cells (PSCs), accounts for up to 90% of the volume of the pancreatic tumor and comprises a complex microenvironment (Neesse et al. 2015). Oncogenic mutations alone are insufficient drivers for disease progression of PDAC, and fibrosis and inflammation are required in addition (Guerra et al. 2007). Notably, PSCs are major contributors to pancreatic fibrosis and tumorigenesis through the production of extracellular matrix (ECM) proteins and secretion of cytokines, such as transforming growth factor β (TGF-β) and interleukin-6 (IL-6) (Wu et al. 2017; Hwang et al. 2008). These factors promote fibrosis, tumor cell proliferation, angiogenesis, immunosuppression, and therapeutic resistance, leading to disease progression (Vonlaufen et al. 2008). The immune infiltrate in PDAC is rife with immunosuppressive elements that support tumorigenesis (Clark et al. 2007; Seifert et al. 2016). Antigen-presenting cells, including M2-polarized tumor-associated macrophages (TAMs) and myeloid-derived suppressor cells (MDSCs), support PDAC progression by inducing adaptive immune suppression (Pylayeva-Gupta et al. 2012; Bayne et al. 2012; Zhu et al. 2014). Intratumoral CD4^+^ Th2 cells and regulatory T cells (Tregs) are associated with reduced survival, whereas cytotoxic CD8^+^ T cells and CD4^+^ Th1 cells mediate tumor protection and are associated with prolonged survival in PDAC (Monte et al. 2011; Fukunaga et al. 2004; Hiraoka et al. 2006). Gamma-delta T cells (γδ T cells), a non-MHC-restricted lymphocyte subset, constitute a central source of immune-suppressive checkpoint ligands and are important regulators of effector T-cell activation in PDAC. Blockade of PD-L1 in γδ T cells enhanced CD4^+^ and CD8^+^ T-cell infiltration and induced tumor protection in murine PDAC (Daley et al. 2016). Furthermore, breast cancer-infiltrating γδ T cells have the capacity to suppress dendritic cell function and, consequently, cytotoxic T-cell activation (Peng et al. 2007). In contrast, several studies in other tumors, including melanoma, renal cell, and colon cancer, suggest that γδ T cells may have antitumoral effects (Gao et al. 2003; Girardi et al. 2001; Lanca and Silva-Santos 2012). Notably, in PDAC, the interaction of γδ T cells with other components of the tumor stroma is unknown. In this study, we found infiltration of γδ T cells in the tumor stroma of human PDAC. PDACs with a high expression of the γδ T-cell-related gene *TRGC2* also had increased expression of several extracellular matrix genes. γδ T cells promoted IL-6 production by PDAC-derived PSCs. Our results suggest that γδ T cells may have a direct effect on PSCs and that γδ T-cell modulation in PDAC may relieve local immunosuppression leading to increased invasion of cytotoxic T cells.

## Materials and methods

### Patient samples

Tissues were obtained from 68 patients with PDAC who underwent surgery at our institution and were consented to a protocol approved by the Ethics Committee of the TU Dresden. A serial section from each specimen was stained with H&E for histologic evaluation. The clinical stages of tumors were determined according to the tumor-node-metastasis (TNM) classification system by the Union For International Cancer Control (UICC; Edition 8). Adjacent non-tumor tissue was used as normal pancreas. Patients’ characteristics are shown in (Table 1).

**Table 1:** Clinicopathological features of PDAC patients in immunohistochemistry cohort

	Total *n* = 68	%
Age		
Median (range)	70 (36–84)	
Gender		
Male	36	52.9
Female	32	47.1
pT		
1	6	8.8
2	43	63.2
3	19	28
4	0	0
pN		
0	26	38.2
1	23	33.8
2	19	28
pM		
0	62	91.2
1	6	8.8
UICC stage		
I	17	25
II	28	41.2
III	17	25
IV	6	8.8
Neoadjuvant treatment		
Yes	3	4.4
No	52	76.5
Unknown	13	19.1

### Immunohistochemistry and immunofluorescence

Frozen tissue sections were rehydrated and blocked [5% goat serum (Sigma-Aldrich), 1% BSA, 1.5 M Tris HCl] for 30 min, as previously described (Seifert et al. 2016). Anti-γδ TCR (Biolegend), anti-α-SMA, and anti-Cytokeratin 19 (both abcam) were applied at 4 °C overnight. Secondary antibodies against Mouse IgG labeled with Alexa Flour 633, Rabbit IgG labeled with Alexa Fluor 488, and Guinea Pig IgG labeled with Alexa Fluor 568 (all Thermofisher) were used. Nuclei were counterstained with 4′,6-diamidino-2-phenylindole (DAPI, Vector Labs) and embedded in Faramount Mounting Medium (Agilent Dako). Images were acquired on a confocal Leica SP5 MP. For immunohistochemistry, anti-γδ TCR was applied for 12 h, followed by incubation with secondary antibodies for 30 min. Purified Mouse IgG1 was used as isotype control. ImmPACT™ DAB Peroxidase (Vector Labs) was used according to the manufacturer’s instructions. Slides were imaged on Invitrogen EVOS FL Auto Imaging System (Thermo Fisher Scientific). Quantification was performed by assessing ten hotspots as high-power fields (HPF; 20 ×) per slide.

### In vitro γδ T-cell/PSC co-culture

Peripheral blood mononuclear cells (PBMCs) were isolated by density centrifugation over Biocoll Separating Solution (Merck). γδ T cells were selected and expanded from PBMCs according to an established protocol by adding zoledronate (5 μM) and IL-2 (1000 IU/mL) to the culture medium (Kondo et al. 2011). Half of the medium was exchanged every 3 days and zoledronate was deprived consequently. After 14 days of culture, the frequency of γδ T cells was assessed by flow cytometry using mAbs directed against CD45 (HI30), CD3 (SK7), CD4 (RPA-T4), CD8 (SK1), and TCR γδ (B1) (all BD Biosciences). Human pancreatic stellate cells (PSCs) were isolated from pancreatic tumor tissue obtained during pancreatic surgery at our institution from patients with resectable pancreatic adenocarcinomas and cultured, as previously published (Vonlaufen et al. 2010). The purity of the PSCs was assessed by morphology and demonstration of αSMA expression. 7.5 × 10^4^ PSCs were plated alone or with expanded γδ T cells (1:2 ratio) in 12-well plates in triplicate. After 48 h, supernatant was harvested and analyzed by cytometric bead array (CBA; BD Biosciences).

### Flow cytometry

Single-cell suspensions were stained with mAbs directed against CD45 (HI30), CD3 (SK7), CD4 (RPA-T4), CD8 (SK1), and TCR γδ (B1, all BD Biosciences). For intracellular staining, cells were stimulated with phorbol 12-myristate 13-acetate (PMA, 50 ng/mL) and ionomycin (750 ng/mL) for 4 h at 37 °C, 5% CO2 in the presence of 1 mg/mL brefeldin A (BD Biosciences). Surface staining was performed, and cells were fixed and permeabilized with the BD Cytofix/Cytoperm Kit and stained for IL-6 (MQ2-6A3, BD Biosciences). Flow cytometry was carried out on the LSR Fortessa flow cytometer (BD Biosciences). Data were analyzed using FlowJo v10 (Treestar, Ashland, OR).

### Cytometric bead array

Supernatant was harvested and analyzed using a cytometric bead array according to the manufacturer’s protocol (BD Biosciences).

### TCGA data analysis

FPKM values from human PDAC samples were obtained from the TCGA Data Portal (https://tcga-data.nci.nih.gov). Of the pancreatic cancer samples from the TCGA database (*n* = 179), we analyzed only PDAC (*n* = 146). A heatmap was created using heatmapper (https://www.heatmapper.ca).

### Statistical analysis

Data are shown as mean ± SEM or median. Unpaired, two-tailed Student’s *t* test or one-way ANOVA comparisons were performed as applicable. GraphPad Prism 8.0 (GraphPad Software, La Jolla, CA) was used. *P* ≤ 0.05 was considered significant.

## Results

### γδ T cells infiltrate human PDAC

**Fig. 1:**
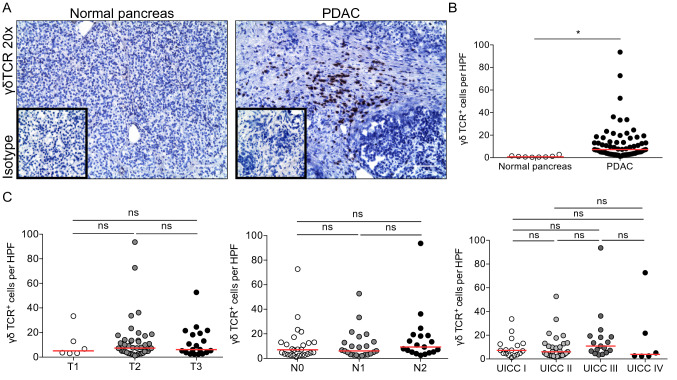
γδ T cells infiltrate human PDAC. **a** Frozen sections of human PDAC (*n* = 68) and adjacent normal pancreas (*n* = 8) were tested for the expression of the γδ T-cell receptor (γδTCR). Representative images are shown. Scale bar, 100 μm. **b** Quantification of γδTCR^+^ cells per high-power field (HPF). **c** Number of γδTCR^+^ cells per HPF correlated with T (left), N (middle), and UICC stages (right). Each point represents data from one patient. Data, median, unpaired *t* test, or one-way ANOVA. *P < 0.05

To investigate the role of γδ T cells in human PDAC, we stained normal pancreas and PDAC samples for the γδ T-cell receptor by immunohistochemistry (Fig. 1a). Normal pancreas had little infiltration of γδ T cells, whereas intratumoral γδ T cells were present in most PDAC patients with a heterogeneous distribution (Fig. 1b). However, the presence of γδ T cells was not associated with tumor size, lymphnode metastasis, and UICC stage (Fig. 1c).

### High γδ T-cell infiltration is associated with fibrosis in PDAC

**Fig. 2:**
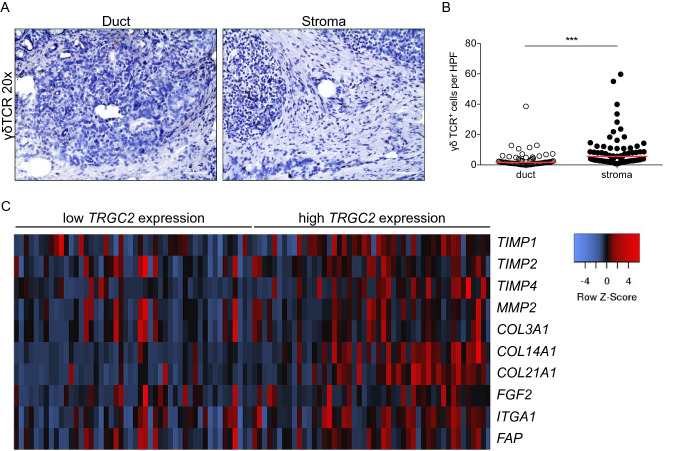
High γδ T-cell infiltration is associated with fibrosis in PDAC. **a** Frozen sections of human PDAC (*n* = 68) were tested for the expression of the γδ T-cell receptor (γδTCR) and analyzed by their intratumoral location (duct vs. stroma). Representative images are shown. Scale bar, 100 μm. **b** Quantification of γδTCR^+^ cells per high-power field (HPF). Each point represents data from one patient. Data, median, unpaired *t* test. ***P < 0.001 (**c**) Heatmap showing low and high tertiles of *TRGC2* expression and indicated extracellular matrix genes in human PDAC samples from the TCGA database. Row, indicated gene; columns; color key indicates row Z-score

γδ T cells were mostly located in the pancreatic tumor stroma, whereas a minor fraction of γδ T cells was present in the ductal area (Fig. 2a, b). We next investigated PDAC samples from the TCGA database with high *TRGC2* expression and found a strong correlation with the expression of several ECM-related genes (Fig. 2c).

### γδ T cells are located in proximity to PSCs in the pancreatic tumor stroma

**Fig. 3:**
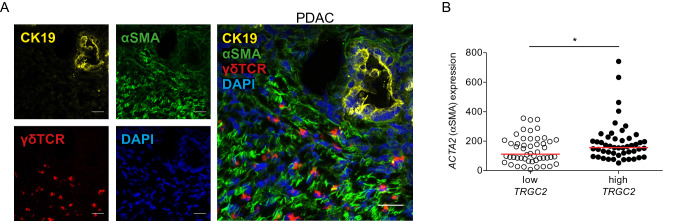
γδ T cells are located in proximity to PSCs in the pancreatic tumor stroma. (**a**) Frozen human PDAC specimens were stained for alpha-smooth muscle actin (αSMA), γδTCR and CK19 by immunofluorescence. Representative image is shown. Scale bar, 25 μm. **b** Correlation between the high and low tertiles of *TRGC2* expression and *ACTA2* expression was tested in human PDAC samples from the TCGA database. Each point represents data from one patient. Data, median, unpaired *t* test. *P < 0.05

Using immunofluorescence staining for the γδ T-cell receptor and alpha-smooth muscle actin (αSMA), a differentiation marker for activated PSCs, we found that γδ T cells were located near PSCs in the pancreatic tumor stroma (Fig. 3a). Consistent with this observation, PDAC samples from the TCGA database with high *TRGC2* expression also had increased *ACTA2* (αSMA) expression compared to samples with low *TRGC2* expression (Fig. 3b).

### γδ T cells activate PSCs and stimulate their IL-6 production

**Fig. 4:**
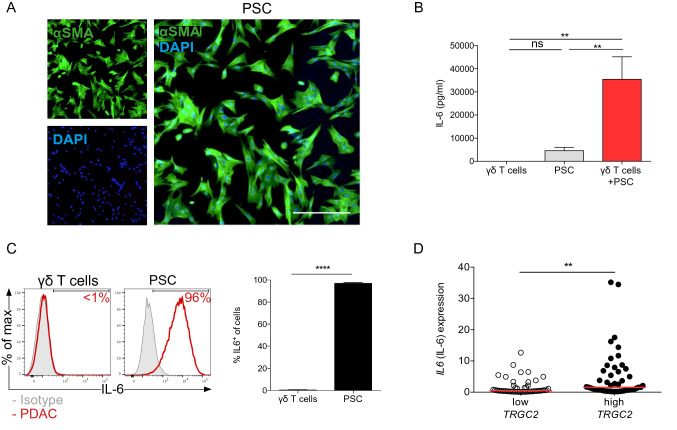
γδ T cells activate PSCs and stimulate their IL-6 production. **a** PSCs were tested for alpha-smooth muscle actin (αSMA) expression by immunofluorescence. Representative image is shown. Scale bar, 25 μm. **b** PSCs (7.5 × 10^4^) were plated alone or together with expanded γδ T cells (1:2 ratio) in 12-well plates and IL-6 expression was measured in the supernatant by cytometric bead array (CBA). **c** γδ T cells and PSCs were tested for IL-6 expression. Representative histograms and quantification are shown. **d** Correlation between the high and low tertiles of *TRGC2* expression and *IL6* expression was tested in human PDAC samples from the TCGA database. Each point represents data from one patient. Data, mean or median, unpaired *t* test, or one-way ANOVA. **P < 0.01, ****P < 0.0001

To further investigate the effect of γδ T cells on PSCs, we co-cultured γδ T cells with PDAC-derived PSCs. PSCs were isolated from pancreatic tumor tissue obtained during surgery from patients with resectable pancreatic adenocarcinomas and their purity was assessed by αSMA expression (Fig. 4a). Using a cytometric bead array (CBA), we measured cytokine production after 48 h. IL-6 production by PSCs was increased after co-culture with γδ T cells compared to the monoculture of PSCs alone (Fig. 4b). Notably, intracellular staining showed that IL-6 was derived from PSCs exclusively and not γδ T cells (Fig. 4c). Further analysis of the TCGA database revealed a significant correlation of *IL6* with high *TRGC2* expression compared to *TRGC2* low PDAC samples (Fig. 4d).

## Discussion

PDAC is characterized by a dense fibrotic tumor stroma that surrounds tumor cells, compromising the efficacy of antitumor therapies and leading to poor patient survival (Watt and Kocher 2013; Feig et al. 2012). γδ T cells regulate effector T-cell activation in PDAC. γδ T-cell depletion was protective against murine PDAC and resulted in increased infiltration, activation, and Th1 polarization of αβ T cells (Daley et al. 2016). In this study, we found the infiltration of γδ T cells in the tumor stroma of human PDAC, particularly in close proximity to PSCs. γδ T-cell infiltration, based on *TRGC2* gene expression, correlated with several ECM genes.

PSCs have been demonstrated to play an important role in fibrogenesis and tumorigenesis in PDAC (Apte et al. 2013). These myofibroblast-like cells secrete ECM proteins as well as many proinflammatory cytokines and are responsible for the desmoplastic reaction. However, little is known about the interaction of PSCs with immune cells in the tumor stroma. Previously, an increased migration of CD8^+^ T cells towards activated, CXCL12-secreting PSCs was shown (Ene-Obong et al. 2013). Additionally, PSC-conditioned media attracted increased numbers of cytotoxic CD8^+^ and CD4^+^ T cells in vitro, suggesting that PSCs are important regulators of immune cell infiltration into the pancreatic tumor stroma. Furthermore, PSCs have been shown to promote differentiation of the MDSC phenotype through IL-6 and suppress T-cell proliferation (Mace et al. 2013).

Immunotherapeutic strategies have failed to improve overall survival for PDAC patients (Brahmer et al. 2012). However, immunotherapy in combination with stromal depletion has led to reduced tumor size and prolonged survival in murine PDAC. While in transgenic *Ptf1a*^*cre/*+^*;LSL-Kras*^*G12D/*+^*;Tgfbr2*^*flox/flox*^ (PKT) mice, deletion of αSMA^+^ myofibroblasts did not enhance gemcitabine efficacy, treatment with anti-CTLA-4 decreased disease acceleration and prolonged survival (Ozdemir et al. 2014). Notably, FAP^+^ stromal cells are the principal source of CXCL12 in PDAC. Blockade of the CXCL12–CXCR4 axis induced T-cell infiltration and enhanced the antitumor effects of anti-CTLA-4 and anti-PD-L1 treatment in murine PDAC (Feig et al. 2013). Currently, therapeutic strategies are being investigated in which stromal depletion is pursued.

Generally, the interaction between immune cells and PSCs in the pancreatic tumor microenvironment has not been well defined. Mast cells have been shown to contribute to PSC proliferation through IL-13 and tryptase, contributing to PDAC development (Ma et al. 2013). Additionally, IL-15 enhanced natural killer cell cytotoxicity towards PSCs in vitro (Audenaerde et al. 2017).

In this study, we found that γδ T cells activated PSCs and stimulated their IL-6 production. In PDAC patients, increased IL-6 serum levels have been correlated with tumor size and the presence of liver metastases (Talar-Wojnarowska et al. 2009; Ebrahimi et al. 2004). Notably, PSC-derived IL-6 directly enhanced STAT3-dependent progression of PanINs towards invasive carcinomas (Nagathihalli et al. 2016). Furthermore, IL-6 promoted PDAC tumorigenesis through downstream activation of the STAT3/SOCS3 signaling pathway (Lesina et al. 2011). In colorectal cancer, IL-6/IL-11-dependent STAT3 activation in cancer-associated fibroblasts promoted tumor development and also correlated with poor prognosis (Heichler et al. 2019). Our study provides evidence that γδ T cells activate PSCs to secrete IL-6, which may, in turn, promote PDAC development and progression. A pre-clinical study indicated that targeting IL-6 may enhance the antitumor efficacy of PD-L1 blockade in PDAC through increased T-cell activation and infiltration (Mace et al. 2018). Furthermore, IL-6 in colorectal cancer and ovarian cancer ascites with a high concentration of IL-6 has been shown to polarize M2 macrophages (Chen et al. 2018; Duluc et al. 2007). Several studies have shown that macrophages play an important role in tumorigenesis depending on their phenotype—M1 macrophages are associated with an inflammatory response and antitumor immunity, whereas M2 macrophages promote angiogenesis and immunosuppression and enhance tumor progression (Sica and Mantovani 2012). In PDAC, M2-polarized macrophages have tumor-promoting effects by releasing immunosuppressive cytokines and inducing Th2 and regulatory T-cell differentiation of CD4^+^ T cells (Liu et al. 2013).

In conclusion, γδ T cells are novel stimuli of PSC secreted IL-6 contributing to their role as critical regulators of immunosuppression and tumor progression in PDAC. Modulation or inhibition of γδ T cells alone or in combination with IL-6-receptor blockade may enhance the efficacy of chemotherapy and immunotherapy in PDAC.
